# Comparison of two different types of hybrid Tibial fixations for anterior cruciate ligament reconstruction: a prospective comparative cohort study

**DOI:** 10.1186/s12891-022-06057-3

**Published:** 2022-12-14

**Authors:** Po-Jen Lai, Chin-Chean Wong, Wen-Pei Chang, Chen-Kun Liaw, Chih-Hwa Chen, Pei-Wei Weng

**Affiliations:** 1 Department of Orthopaedics, Shuang Ho Hospital, Taipei Medical University, New Taipei City, 235 Taiwan; 2 Department of Orthopaedics, School of Medicine, College of Medicine, Taipei Medical University, Taipei City, 110 Taiwan; 3 Graduate Institute of Biomedical Materials and Tissue Engineering, College of Biomedical Engineering, Taipei Medical University, Taipei City, 110 Taiwan; 4 Research Center of Biomedical Devices, Taipei Medical University, Taipei, 11031 Taiwan; 5 International Ph.D. Program for Cell Therapy and Regenerative Medicine, College of Medicine, Taipei Medical University, Taipei, 11031 Taiwan; 6 Non-Invasive Cancer Therapy Research Institute of Taiwan, Taipei, 10489 Taiwan; 7 Department of Nursing, Shuang Ho Hospital, Taipei Medical University, New Taipei City, Taiwan; 8 School of Nursing, College of Nursing, Taipei Medical University, Taipei, Taiwan; 9 International Ph.D. Program in Biomedical Engineering, College of Biomedical Engineering, Taipei Medical University, Taipei, 11031 Taiwan

**Keywords:** Anterior cruciate ligament reconstruction, Tunnel widening, Tunnel enlargement, Hamstring graft, Hybrid fixation, Adjustable suspensory device

## Abstract

**Background:**

Previous studies have compared different kinds of fixations for anterior cruciate ligament reconstruction. Nevertheless, there is no optimal method to date. To the best of authors’ knowledge, there is no article discussing the combination of adjustable suspensory device and interference screw for hybrid tibial fixation.

**Methods:**

In total, 66 patients (*n* = 34, adjustable suspensory device and interference screw; *n* = 32, cortical screw and interference screw) were analyzed. Their International Knee Documentation Committee score and Tegner activity level scale were evaluated before and after a 2-year follow-up. The Single Assessment Numeric Evaluation score was evaluated after a 2-year follow-up. Physical exams such as range of motion, anterior knee pain (VAS > = 3) and Lachman test were assessed before and at least 12 months after surgery. To evaluate tunnel widening, anteroposterior and lateral view radiography was conducted 1 day and at least 12 months after surgery. A more than 10% change was considered tibial tunnel widening. Mann–Whitney *U* test, independent *t* test, paired *t* test, Fisher’s exact test and chi-squared test were used to compare the variables. Linear and logistic regression models were applied to adjust for potential confounders.

**Results:**

No variable except gender (*P* = 0.006) showed significant difference with regard to demographic data. After adjustment, there was no statistically significant difference between the groups regarding post-operative physical exams. Patients who used adjustable suspensory device and interference screw had lower post-operative Single Assessment Numeric Evaluation score (adjusted β − 8.194; *P* = 0.017), Tegner activity level scale (adjusted β − 1.295; *P* = 0.001) and pre-operative degrees of knee flexion (adjusted β − 2.825; *P* = 0.026). Less percentage of tunnel widening in the lateral view of radiographs was seen in patients in group of adjustable suspensory device and interference screw (adjusted β − 1.733; *P* = 0.038). No significant difference was observed in the anteroposterior view of radiographs (adjusted β − 0.667; *P* = 0.26).

**Conclusion:**

In these 66 patients, we observed less tibial tunnel widening and lower post-operative functional scores in the group of adjustable suspensory device and interference screw. Both groups displayed similar outcomes of physical exams as well as improvement after operation. The proposed method may become an alternative option. Nonetheless, the quality of our study is still limited, and thus further studies are warranted to determine the efficacy and further application.

**Trial registration:**

Joint Institutional Review Board of Taipei Medical University, Taipei, Taiwan (No: N201805094).

**Study design:**

Prospective comparative cohort study; Level of evidence, II.

## Introduction

In the United States, more than 120,000 ACL ruptures were reported every year [1], and the annual incidence of ACL rupture was approximately 68.8 per 100,000 person-years [2]. The increasing participation in sports and recreational activities has increased the risk of ACL rupture at present. Consequently, anterior cruciate ligament (ACL) reconstruction has become one of the most frequently performed orthopedic operations worldwide. Although ACL reconstruction is routinely performed, the optimal choice of fixation remains controversial.

Several methods for ACL fixation are available, such as interference screw, fixed or adjustable suspensory devices, and hybrid fixation [3]. Nonetheless, no method has been shown superiority to other methods in terms of both graft strength and clinical results such as functional scores and physical examination. All fixation methods have their own pros and cons, and previous studies have attempted to determine the optimal choice of method for ACL reconstruction. In a previous meta-analysis, Browning et al. compared suspensory devices to interference screws and observed that suspensory devices resulted in a better overall knee stability and less graft rupture [4]; by contrast, a recent systematic review and meta-analysis by Fu et al. reported no significant difference in knee stability and graft strength between suspensory devices and interference screws, except for less tibial tunnel widening (TW) when suspensory devices were used [5]. Similarly, clinical outcomes such as functional scores and physical examinations showed no significant difference when either of the methods was used [4, 5]. With regard to suspensory devices, whether fixed or adjustable suspensory devices should be used remains debatable. Studies that involved the comparison of fixed and adjustable suspensory devices showed similar knee laxity when either type of device was used, and no significant difference was noted between the devices in terms of clinical outcomes [6, 7].

As there was no optimal method of choice for single-mode fixation in ACL reconstruction, hybrid fixation, which combined the advantages of both methods, was proposed. Most of the hybrid tibial fixation techniques involve mounting of a cortical screw post in addition to an intra-tunnel interference screw to increase the strength of the graft. The tibial side was frequently considered the weak point of ACL reconstruction owing to the less dense metaphyseal bone and more parallel vector of force at this side [8]. Nevertheless, many studies on hybrid fixation have been published and Balazs et al. reported that hybrid tibial fixation afforded better initial graft strength and less knee laxity compared with single-mode fixation. Yet, no significant difference in clinical outcomes was observed after a follow-up of 1 to 3 years between patients who underwent hybrid tibial fixation and those who underwent single-mode fixation [9].

To determine the optimal fixation for ACL reconstruction, we combined adjustable suspensory devices and interference screw (ASIS) on the tibial side. To the best of our knowledge, this modified technique has not been discussed and compared with other types of fixations to date. We hypothesized that compared with hybrid tibial fixation using a cortical screw post along with the interference screw(CSIS), our modified method could yield similar clinical outcomes in terms of the Lachman test, range of motion (ROM), International Knee Documentation Committee score (IKDC), Single Assessment Numeric Evaluation (SANE) score, and Tegner activity level scale and fewer commonly reported complications such as tibial TW and anterior knee pain (VAS > = 3) [10–13].

## Materials and methods

### Patients

This study was approved by the Joint Institutional Review Board of Taipei Medical University, Taipei, Taiwan (No: N201805094). We recruited consecutive patients who had undergone arthroscopic ACL reconstruction with single-bundle and quadrupled hamstring autograft from July 2015 to March 2018. These patients had received either CSIS fixation or ASIS fixation conducted by single surgeon, Dr. Pei-Wei Weng. Patients were assigned to the ASIS or CSIS group according to their own choice after our explanation. Initially, 108 patients were included. Patients with concomitant posterior cruciate ligament or medial collateral ligament injuries more than grade II, those aged less than 18 years, those who underwent revision ACL reconstruction, those with avulsion fracture, those underwent meniscal repair and those with contralateral knee injuries were excluded.

### Operative technique

An exploratory arthroscopy was conducted anteromedially and anterolaterally to assess the presence of any additional lesions such as a meniscus tear, cartilage damage, or loose bodies.

**Fig. 1:**
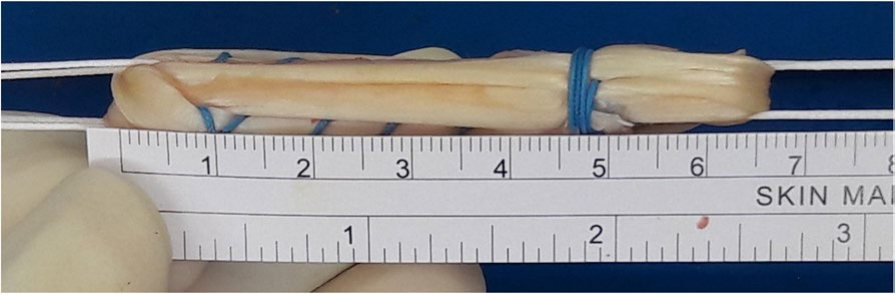
Single-bundle hamstring graft used in anterior cruciate ligament reconstruction

**Fig. 2:**
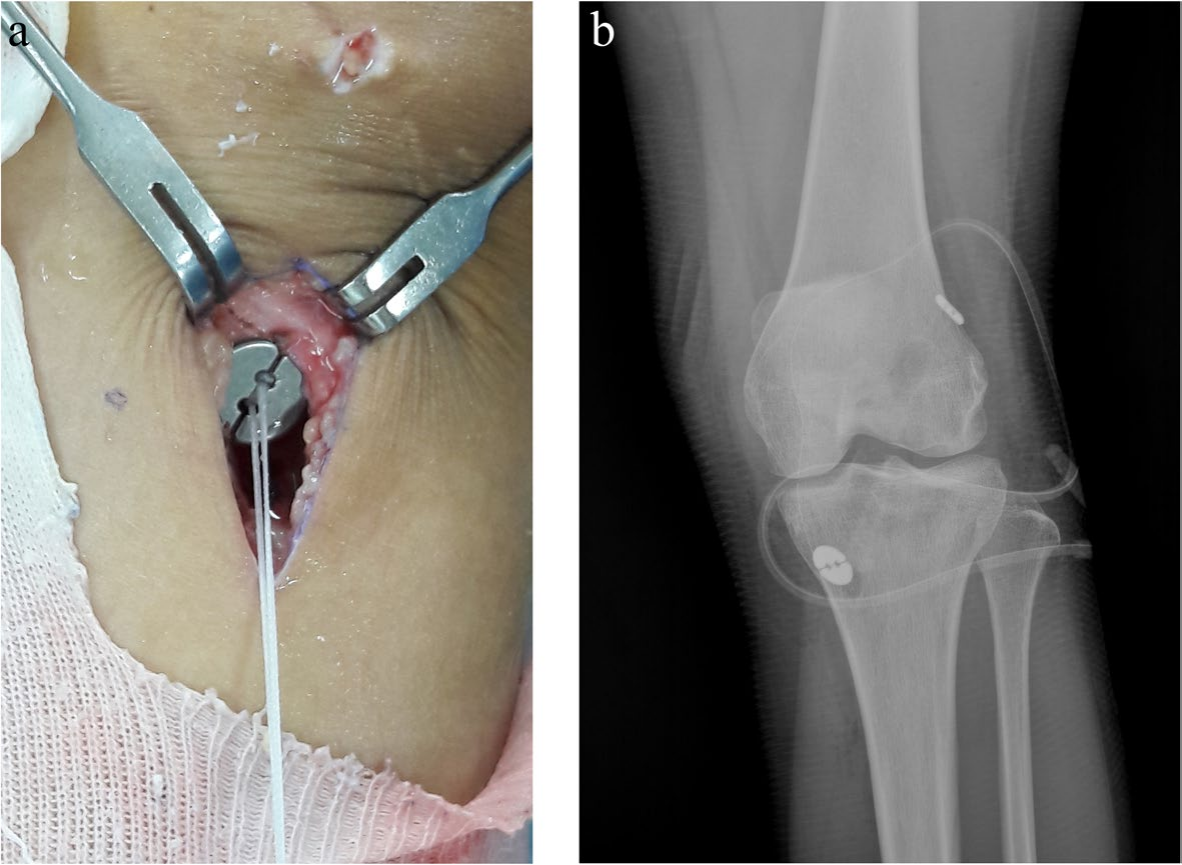
**a** Modified hybrid fixation with TightRope ABS implants (Arthrex) on the tibial side. **b** Postoperative radiograph revealing application of suspensory devices in modified hybrid tibial fixation

**Fig. 3:**
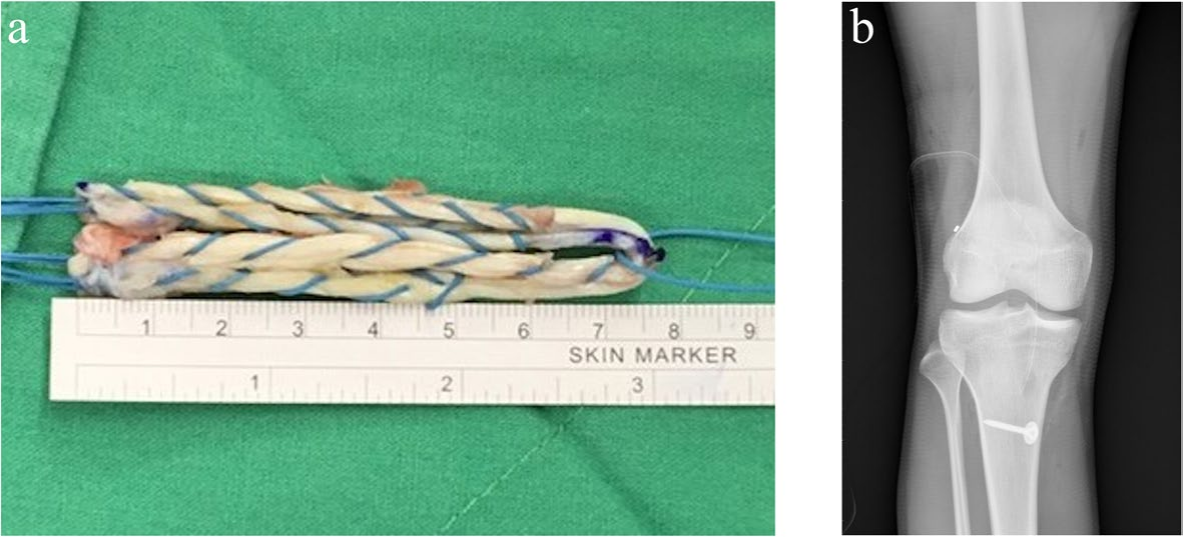
**a** Whipstitches at tibial ends of the graft with nonabsorbable sutures (No. 5 Ethibond) for later fixation with a cortical screw post. **b** Postoperative radiograph showing traditional hybrid tibial fixation using a bioabsorbable interference screw and cortical screw post

A longitudinal skin incision measuring approximately 3 cm was then made on the anteromedial tibial surface at the level of the pes anserinus. The semitendinosus as well as gracilis tendons were then harvested from their distal insertion by a closed tendon stripper. The graft was then folded twice to make it four stranded to reach a proper length of around 65–75 mm (Fig. 1) while ensuring that a 1-minute pretension was performed; the diameter of the hamstring graft ranged from 8 to 11 mm. Finally, TightRope RT implants (Arthrex, Inc., Naples, Florida, USA) for the femoral side and TightRope ABS implants (Arthrex) for the tibial side were installed onto the hamstring graft of patients in ASIS group (Fig. 2a and b). For those in CSIS group, TightRope RT implants (Arthrex) were connected to the femoral side and whipstitches were placed at the tibial ends of the tendon with nonabsorbable sutures (No. 5 Ethibond) for later fixation with the cortical screw post (Fig. 3a and b) onto the proximal tibia.

Once the preparation was done, we started to establish the femoral socket and tibial tunnel. Under maximal knee flexion (≥130 degrees of flexion) on the table, we drilled the femoral socket through the anteromedial portal with inside-out approach, attempting to reach the anatomical anteromedial bundle insertion site at the lateral femoral condyle. The femoral socket was positioned at 10 o’clock for the right knees and 2 o’clock for the left knees. We drilled firstly using a 4.5 mm drill to create path for the TightRope RT implants (Arthrex, Inc., Naples, Florida, USA). Another reamer which was compatible to the diameter of graft was then applied to create a proper depth of femoral socket (approximately 20–25 mm) for the graft. For the tibial tunnel, the transtibial technique was performed. Under assistance of alignment device modulated to 47.5° to 52.5°, a drill pin was passed through the center of the ACL footprint. The tibial tunnel was then adjusted to match the diameter of the graft by using reamers that were compatible to the size of grafts.

Subsequently, the graft was placed into the joint. The passing suture was employed so that the TightRope sutures and TightRope button could be passed through. Following 10 sets of flexion and extension, the tibial-sided graft was secured with a bioabsorbable interference screw in the tibial tunnel for patients in both the groups. Lastly, the Tightrope ABS was firmly tied in the ASIS group and the cortical screw post was placed in the CSIS group.

### Rehabilitation

After the operation, quadriceps and active ROM exercises in sitting or lying positions without weight-bearing were initiated as soon as possible (1 day postoperatively). Weight-bearing training was also initiated under assistance of a knee brace (starting from full extension and flexed 10° weekly thereafter) for 2 months. The majority of the patients could resume jogging approximately 6 months postoperatively.

### Assessment of clinical outcomes

Physical exams were performed before as well as at least 12 months after the operation at the outpatient clinic. ROM measurement and Lachman test were conducted by experienced orthopedic surgeons. Pivot shift test were performed before operation at operation room under anesthesia [14]. As for the grading of the Lachman test, we did not apply measurement instrument such as KT-1000. Some results were equivocal and thus we graded them with “I-II” and “II-III”. The grades not higher than grade II were considered stable with firm end-feel; on the contrary, the grades higher than grade II were deemed unstable with no firm end-feel. As for ROM, we had measured the flexion and extension of injured knee before and after the operation. The examiners were blinded with regard to the operative methods. In terms of functional scores, we had recorded IKDC score and Tegner activity level scale prior to the operation. Subsequently, subjective IKDC score, SANE score and Tegner activity level scale were documented during the patients’ visit to the clinic or via telephonic inquiry 2 years after the operation.

**Fig. 4:**
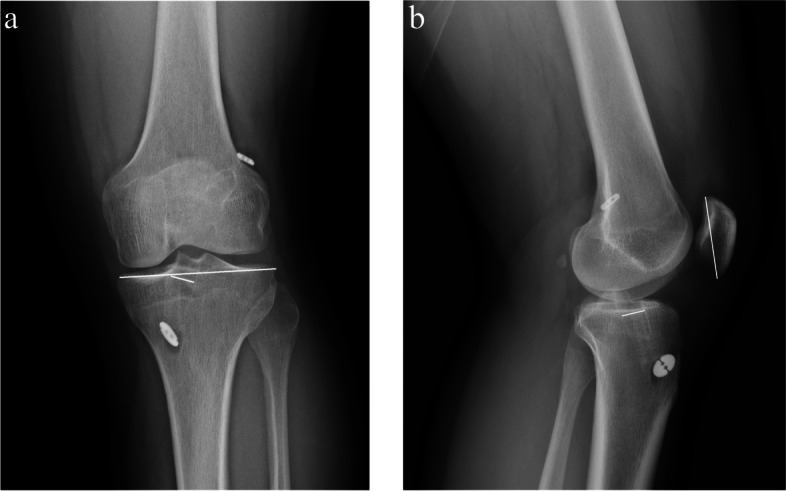
**a** Measurement of percentage of tunnel widening in anteroposterior view of radiographs. **b** Measurement of percentage of tunnel widening in lateral view of radiographs

With regard to tibial TW assessment, all patients underwent anteroposterior (AP) and lateral (LAT) view radiography on the next day of the operation and at least at the 1-year follow-up. The interval between two evaluations was decided based on previous studies [15–17]. To determine the width of the tibial tunnel, we measured the diameter between two sclerotic edges approximately 3 mm below the tibial plateau [16]. Further, to compare with the width of the tunnel on Day 1 after the operation, the diameter was divided by the maximal width of the proximal tibia in the AP view and that of the patella in the LAT view (Fig. 4a and b). A minimal enlargement of 10% in the tunnel diameter is defined as TW, and this definition was also used in our study [15, 18]. With regard to tunnel measurement, we performed test-retest reliability (repeatability test) as tunnel measurement was carried out by one single resident. Measurements were performed separately a month apart. Pearson correlation coefficient more than 0.8 indicated high correlation while less than 0.4 indicated low correlation. The correlation coefficient from each variable ranged from 0.903 to 0.981 in this study, representing good reliability.

The difference between the period of follow-up was because of how the data was obtained. Both physical examinations and TW measurement required face-to-face assessment, while the functional scores could be acquired via telephonic inquiry. Those who did not show up at the outpatient clinic 2 years after the operation could still provide functional scores to us remotely.

### Statistical analysis

Statistical analysis was conducted using IBM SPSS Statistics for Mac OS (IBM Corporation, Armonk, NY, USA) and R 4.1.0 (R Core Team, Vienna, Austria). First, we checked whether our data showed normal distribution. As both groups comprised fewer than 50 patients, the Shapiro–Wilk test was used. The *P* values for each continuous variable from both the groups were interpreted while only the IKDC scores revealed a significant difference between the groups (*P* <  0.05), indicating that all continuous variables except for IKDC score displayed normal distribution. We then analyzed the statistical differences in all continuous variables except for the IKDC score by using the independent *t* test, and the IKDC score was analyzed using the Mann–Whitney *U* test instead. The chi-squared test and the Fisher’s exact test were used for categorical variables. Lastly, linear and logistic regression models were applied to adjust for potential confounders. The level of significance was set at *P* <  0.05.

## Results

### Patients

**Fig. 5:**
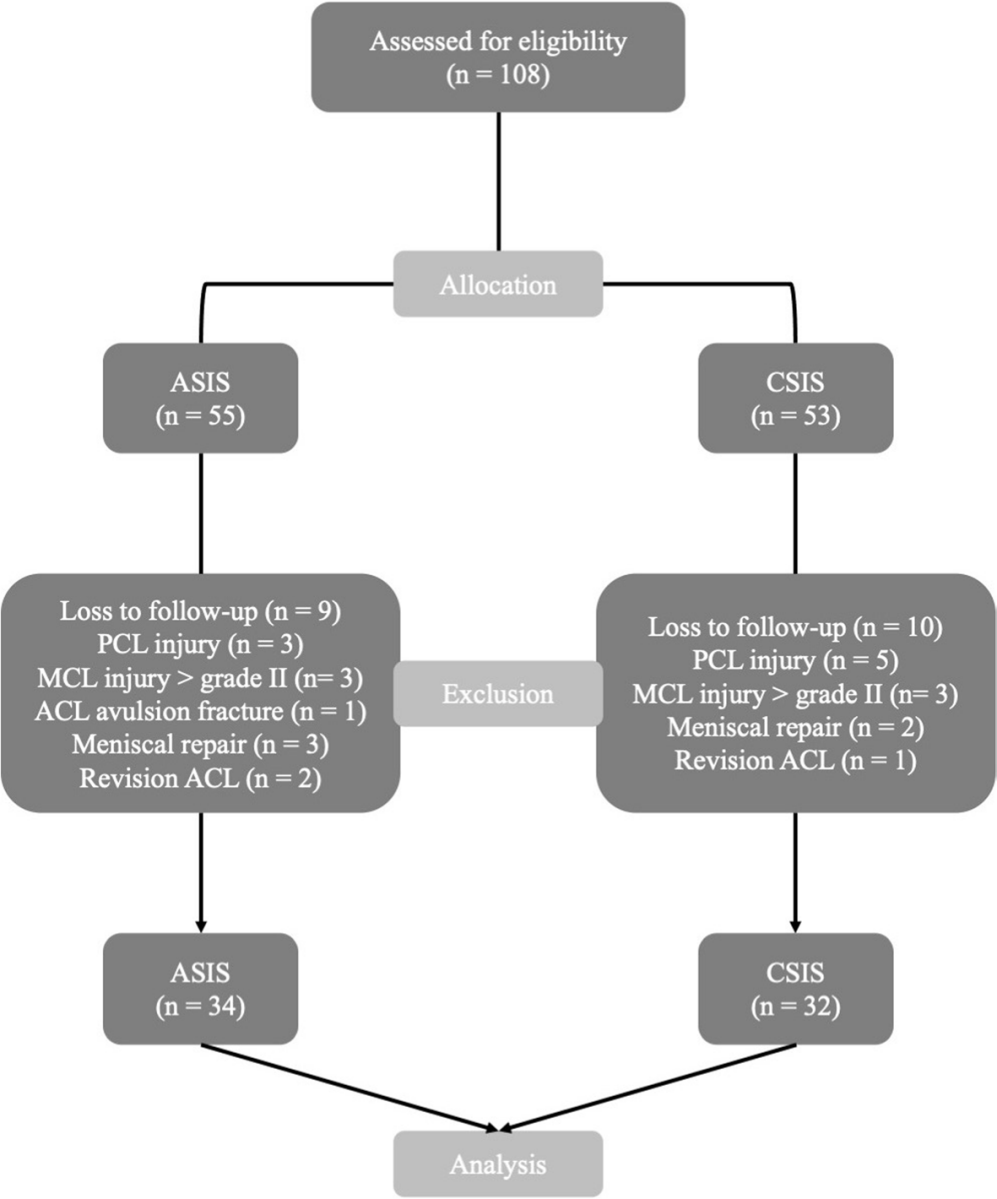
The flow of participants throughout the research

A total of 108 patients who prepared to undergo ACL reconstruction with single-bundle and quadrupled hamstring graft were assessed. A flowchart regarding the allocation is shown in Fig. 5. Among these patients, 55 patients had chosen ASIS group whereas 53 patients had decided to join CSIS group in the beginning. Several patients from the groups were excluded owing to the following reasons: eight patients had simultaneous posterior cruciate ligament injury, six patients had concomitant medial collateral ligament injury greater than grade II, one patient had ACL avulsion fracture, five patients had undergone meniscal repair and three patient was undergoing revision ACL reconstruction. Further, nine patients from the ASIS group and ten patients from the CSIS group were lost to follow-up. Eventually, 34 patients in the ASIS group and 32 patients in the CSIS group were enrolled in the study.

The demographic data of the patients are presented in Table 1. The preoperative pivot shift test was conducted under anesthesia to lower the error related to muscle tone, and the results were categorized as positive and negative. Whereas the pivot shift test after operation was carried out in outpatient clinic, fear for the exam and the resistance created by muscle tone under awake status could affect the accuracy of the examination. Thus, we excluded the post-operative results of the pivot shift test. No significant difference was observed between the groups with regard to age (*P* = 0.393), duration of injury (*P* = 0.507), surgical site (*P* = 0.833), simultaneous meniscus injury (*P* = 0.661), preoperative pivot shift test (*P* = 0.852) and type of injury (*P* = 0.855). With regard to meniscal injury, there was no significant difference between the groups. These patients had undergone partial meniscectomy if necessary. As for the type of injury, sporting activities were the main reason of ACL injury in both groups. Accident had a role as well since motorcycle-related traffic accident was a major issue in this region. On the contrary, the distribution of gender significantly differed between the groups (*P* = 0.006). The ASIS group consisted of 27 men and 7 women, and the CSIS group consisted of 15 men and 17 women. The proportion of female in the CSIS group was higher and might act as confounder. Thus, we had conducted linear and logistic regression for adjustment. The data of CSIS group were used as reference and the calculated results are presented throughout Tables 2, 3 and 4.

**Table 1:** Demographic data of patients*

	ASIS group	CSIS group	*P* value
Number of cases	34	32	
Age	34.91 ± 9.05	33.09 ± 8.05	0.393 ^‡^
Gender (Male/Female)	27/7	15/17	0.006 ^œ^
Duration of injury (weeks)	19.5 ± 8.7	18 ± 9.6	0.507 ^‡^
Surgical site (R’t/L’t)	14/20	14/18	0.833 ^œ^
Pre-OP pivot shift test (Positive)	22 (64.7%)	20 (62.5%)	0.852 ^œ^
Meniscus injury (Positive)	23 (67.6%)	20 (62.5%)	0.661 ^œ^
Type of injury			0.855 ^¥^
Work-related	3 (9%)	3 (9.4%)	
Sports	28 (82%)	24 (75%)	
Traffic accident	3 (9%)	4 (12.5%)	
Other	0	1 (3%)	

* Continuous values are documented as mean and standard deviation unless there is other indication; ^‡^ Independent T test; ^œ^ Chi-square test; ^¥^ Fisher’s exact test

**Table 2:** Physical exams

		ASIS group (*n* = 34)	CSIS group (*n* = 32)	*P* value	Adjusted β / OR (95% CI)	Adjusted *P* value
Range of motion	Flexion, pre-OP	126.03 ± 5.47	128.12 ± 3.97	0.081 ^œ^	−2.825 (− 5.298, − 0.352)	0.026 ^‡^
	Flexion, post-OP	137.79 ± 3.06	138.59 ± 4.06	0.37 ^œ^	−1.056 (− 2.934, 0.823)	0.27 ^‡^
	Extension, pre-OP	−0.47 ± 1.48	−0.41 ± 1.54	0.86 ^œ^	0.229 (− 0.537, 0.995)	0.55 ^‡^
	Extension, post-OP	−1.29 ± 1.38	−1 ± 1.39	0.39 ^œ^	−0.293 (− 1.024, 0.438)	0.43 ^‡^
Anterior knee pain	Positive	4 (11.7%)	11 (34.4%)	0.028 *	0.34 (0.081, 1.245)	0.11 ^+^
Lachman test	Pre-OP	2.41 ± 0.34	2.5 ± 0	0.14 ^œ^	−0.097 (− 0.225, 0.03)	0.13 ^‡^
	Post-OP	0.91 ± 0.36	0.97 ± 0.22	0.44 ^œ^	−0.053 (− 0.211, 0.104)	0.5 ^‡^

* Chi-square test; ^œ^ Independent T test; ^‡^ Linear regression; ^+^ Logistic regression; *CI* confidence interval

**Table 3:** Functional scores

		ASIS group (*n* = 34)	CSIS group (*n* = 32)	*P* value	Adjusted β (95% CI)	Adjusted *P* value ^‡^
IKDC score	Pre-OP	47.97 (7.79)	49.19 (7.06)	0.51^œ^	−2.347 (−6.181, 1.487)	0.23
	Post-OP	70.38 ± 6.358	71.66 ± 6.434	0.317 ^œ^	− 1.944 (− 5.286, 1.398)	0.25
Tegner activity level scale	Pre-OP	3.18 ± 1.47	3.41 ± 1.29	0.5 *	− 0.57 (− 1.254, 0.114)	0.1
	Post-OP	4.15 ± 1.69	4.94 ± 1.56	0.053*	−1.295 (−2.068, − 0.522)	0.001
SANE score	Post-OP	76.91 ± 12.43	82.97 ± 13.55	0.063*	−8.194 (− 14.852, − 1.536)	0.017

^œ^ Mann-Whitney U test; * Independent T test; ^‡^ Linear regression

**Table 4:** Difference in clinical outcomes before and after the operation

	ASIS group (*n* = 34)	*P* value ^œ^	CSIS group (*n* = 32)	*P* value ^œ^
Tegner diff.	0.97 ± 0.63	< 0.001	1.53 ± 0.76	< 0.001
IKDC diff.	22.38 ± 6.33	< 0.001	22.47 ± 3.14	< 0.001
ROM (flexion) diff.	11.76 ± 4.91	< 0.001	10.47 ± 2.33	< 0.001
ROM (extension) diff.	−0.82 ± 1.59	0.005	− 0.59 ± 2.01	0.11
Lachman diff.	− 1.5 ± 0.51	< 0.001	−1.53 ± 0.22	< 0.001

* diff. = difference; ^œ^ paired T test

### Clinical evaluation

The results of the physical exams are presented in Table 2. We showcased the result of Lachman test by altering the grade into continuous variable. For instance, grade I represented the score of 1. The mean of pre-operative Lachman test of ASIS group was 2.41 ± 0.34, while it of the CSIS group was 2.5 ± 0. No significant difference was observed in the results of the Lachman test between the groups (*P* = 0.14). All patients had undergone magnetic resonance imaging some or the other time before ACL reconstruction. After at least 1-year follow-up, we had evaluated the test during follow-up at outpatient department. The mean score of post-operative Lachman test of ASIS group was 0.91 ± 0.36, whereas it of CSIS group was 0.97 ± 0.22. No significant difference was observed as well between the two groups at the 1-year follow-up (*P* = 0.44). After adjustment for gender, the adjusted β for pre-operative results was − 0.097 (CI -0.225, 0.03; *P* = 0.13), while it of post-operative results was − 0.053 (CI -0.211, 0.104; *P* = 0.5). There was no significant difference.

ROM was documented before operation and at least 1 year after the operation. We had documented degrees of both flexion and extension of the injured knee, and the data are shown in Table 2. With regard to degrees of knee flexion, the pre-operative result of ASIS group was 126.03 ± 5.47, while it of the CSIS group was 128.12 ± 3.97. No significant difference was observed between the groups (*P* = 0.081). The post-operative result of ASIS group was 137.79 ± 3.06, whereas it of CSIS group was 138.59 ± 4.06. There was no statistical significance (*P* = 0.37). After adjustment for gender, the adjusted β for pre-operative results was − 2.825 (CI -5.298, − 0.352; *P* = 0.026), whereas it of post-operative results was − 1.056 (CI -2.934, 0.823; *P* = 0.27). Statistical significance was observed in the degrees of knee flexion before operation after adjustment. As for the degrees of knee extension, the pre-operative result of ASIS group was − 0.47 ± 1.48, while it of CSIS group was − 0.41 ± 1.54. The negative value stands for hyperextension over the neutral point. There was no significant difference between the groups (*P* = 0.86). Further, the post-operative result of ASIS group was − 1.29 ± 1.38, whereas it of CSIS group was − 1 ± 1.39. No significant difference was observed (*P* = 0.39). Adjustment for gender was conducted as well and the adjusted β for pre-operative results was 0.229 (CI -0.537, 0.995; *P* = 0.55), while it of post-operative results was − 0.293 (CI -1.024, 0.438; *P* = 0.43). No significant difference between the groups was observed.

The analysis of anterior knee pain is presented in Table 2. Among the 34 patients in ASIS group, 4 showed anterior knee pain, whereas among the 32 patients in CSIS group, 11 showed anterior knee pain at least 1 year after the operation. Significant difference was observed in the incidence of anterior knee pain between the groups (*P* = 0.028). Adjustment for gender was conducted. The adjusted odds ratio was 0.34 (CI 0.081, 1.245; *P* = 0.11). Unlike the crude data, no statistical significance was observed.

With regard to functional scores, IKDC score and Tegner activity scale were evaluated before and 2 years after the operation, while SANE score was only assessed 2 years after the operation. The results of these functional scores are shown in Table 3. Before the operation, mean total IKDC score of ASIS group was 47.97 (7.79), while it of CSIS group was 49.19 (7.06). No significant difference was observed (*P* = 0.51). Post-operatively, mean total IKDC score of the ASIS group was 70.38 ± 6.358 and that of the CSIS group was 71.66 ± 6.434. No significant difference was observed between the two groups (*P* = 0.317). After adjustment, the adjusted β for pre-operative results was − 2.347 (CI -6.181, 1.487; *P* = 0.23), whereas it of post-operative results was − 1.944 (CI -5.286, 1.398; *P* = 0.25). We did not find significant difference. As for SANE score, the post-operative result in the ASIS group was 76.91 ± 12.43 and that in the CSIS group was 82.97 ± 13.55. No significant difference was observed between the two groups (*P* = 0.063). Adjustment for gender was conducted as well and the adjusted β was − 8.194 (CI -14.852, − 1.536; *P* = 0.017). Contrast to the crude data, statistical significance was observed. Concerning the Tegner activity level scale, the pre-operative mean score of the ASIS group was 3.18 ± 1.47 and that of CSIS group was 3.41 ± 1.29. No significant difference was observed (*P* = 0.5). Post-operatively, mean score of the ASIS group was 4.15 ± 1.69 and that of the CSIS group was 4.94 ± 1.56. No significant difference was observed between the groups (*P* = 0.053). We had carried out adjustment and the adjusted β for pre-operative results was − 0.57 (CI -1.254, 0.114; *P* = 0.1), whereas it of post-operative results was − 1.295 (CI -2.068, − 0.522; *P* = 0.001). Significant difference was observed in the post-operative results.

Additionally, we had compared the difference of these functional scores and clinical outcomes before and after the operation in both groups separately. The results are shown in Table 4. Regarding the functional scores, the difference of Tegner activity level scale was 0.97 ± 0.63 (*P* <  0.001) in ASIS group, while it of CSIS group was 1.53 ± 0.76 (*P* <  0.001). The difference of IKDC score in ASIS group was 22.38 ± 6.33 (*P* <  0.001), whereas it of CSIS group was 22.47 ± 3.14 (*P* <  0.001). Statistical significance was observed. As for the clinical outcomes, the difference of ROM and Lachman within the group were analyzed. The difference of knee flexion was 11.76 ± 4.91 (*P* <  0.001) in ASIS group, while it of CSIS group was 10.47 ± 2.33 (*P* <  0.001). Further, the difference of knee extension was − 0.82 ± 1.59 (*P* = 0.005) in ASIS group, whereas it of CSIS group was − 0.59 ± 2.01 (*P* = 0.11). Significant difference was only observed in the ASIS group. Lastly, the difference of Lachman test in ASIS group was − 1.5 ± 0.51 (*P* <  0.001), while it of CSIS group was − 1.53 ± 0.22 (*P* < 0.001). Statistical significance was observed in both groups.

### Images

**Fig. 6:**
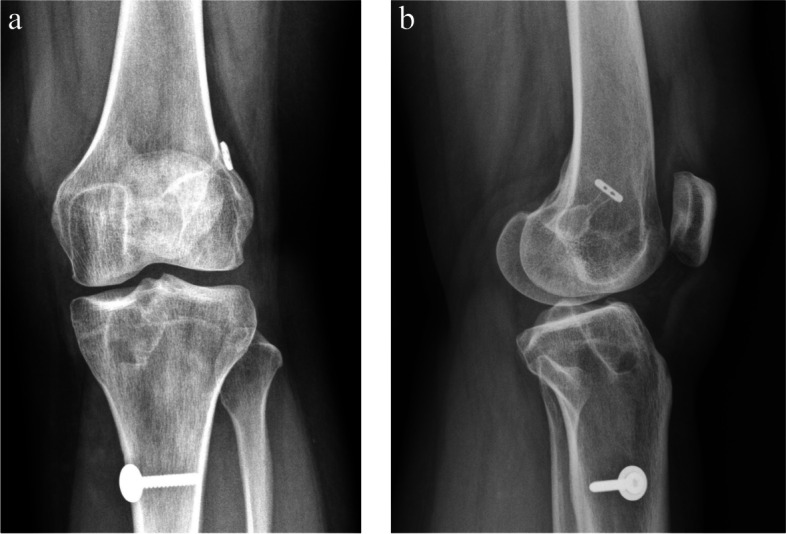
**a** Anteroposterior view of radiograph of CSIS hybrid tibial fixation at least 1 year after operation. **b** Lateral view of radiograph of CSIS hybrid tibial fixation at least 1 year after operation

**Fig. 7:**
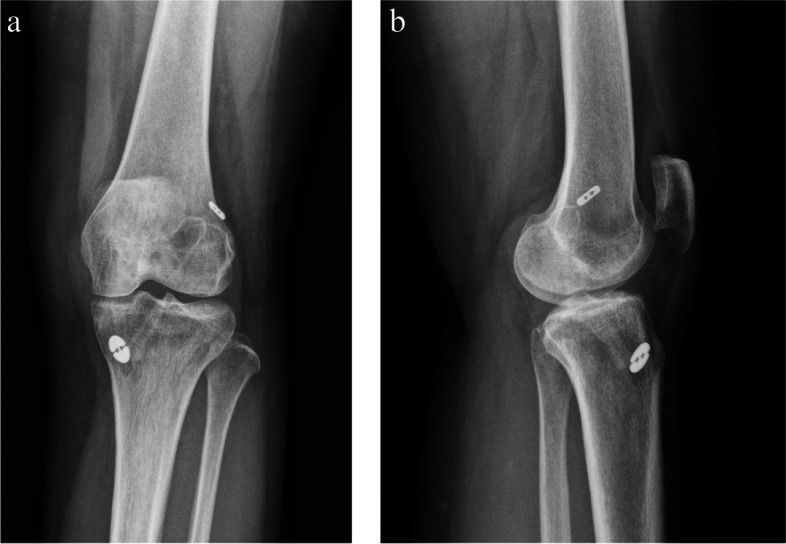
**a** Anteroposterior view of radiograph of ASIS hybrid tibial fixation at least 1 year after operation. **b** Lateral view of radiograph of ASIS hybrid tibial fixation at least 1 year after operation

We compared the percentage TW in the AP and LAT view of radiographs. The data are presented in Table 5 and Figs. 6a, b and 7a, and b. With regard to the percentage of TW in the AP view, no patient from either group showed more than 10% TW during the follow-up period of at least 1 year after surgery (*P* = 1). The mean percentage TW in the ASIS group was 1.923 ± 2.19 and that in the CSIS group was 2.65 ± 2.25 at the 1-year follow-up. No significant difference was observed between the groups even at 1 year after the operation (*P* = 0.188). After adjustment for gender, the adjusted β was − 0.667 (CI -1.835, 0.501; *P* = 0.26). No significant difference was observed either.

**Table 5:** Tunnel widening

		ASIS group (*n* = 34)	CSIS group (*n* = 32)	*P* value	Adjusted β (95% CI)	Adjusted *P* value ^‡^
AP view	> = 10%/ < 10%	0/34	0/32	1 ^*β*^		
	Percentage TW change	1.923 ± 2.19	2.65 ± 2.25	0.188 *	−0.667 (− 1.835, 0.501)	0.26
LAT view	> = 10%/ < 10%	0/34	4/28	0.033 ^œ^		
	Percentage TW change	2.586 ± 3.25	4.364 ± 2.92	0.023 *	−1.733 (− 3.363, −0.103)	0.038

* Independent T test; ^œ^ Chi-square test; ^*β*^ Fisher’s exact test; ^‡^ Linear regression; Percentage TW change = A -B (A = percentage value of TW at least 1 year post-OP; B = percentage value of TW at 1 day post-OP)

As for the TW in LAT view, none of the patients in the ASIS group showed a TW greater than 10% while four patients in the CSIS group showed more than 10% TW during follow-up at least 1 year after the operation. Significant differences were observed between the two groups with regard to the incidence of percentage TW in the LAT view (*P* = 0.033). The mean percentage TW change in the ASIS group was 2.586 ± 3.25 and that in the CSIS group was 4.364 ± 2.92. A significant difference was observed between the groups (*P* = 0.023). Adjustment for gender was conducted as well and the adjusted β was − 1.733 (CI -3.363, − 0.103; *P* = 0.038). Statistical significance was observed.

## Discussion

To the best of our knowledge, this is the first study to investigate the outcomes of ASIS for hybrid tibial fixation in ACL reconstruction. After adjustment for gender, a major highlight of this study was that patients in ASIS group showed less tibial tunnel widening in the LAT view radiographs compared with patients in CSIS group. Meanwhile, we noticed the post-operative functional scores were lower in ASIS group which was different from our hypothesis. Concerning physical exams and anterior knee pain, both groups showcased similar results.

In terms of development of TW, it was considered to be multifactorial in previous studies. Micromotion between the graft and bone interface, early rehabilitation, synovial fluid infiltration, selection of grafts and misplaced graft could all lead to a higher incidence of TW [18–23]. The type of fixation was considered one of the most important factors for tibial TW, and thus previous studies have compared all types of fixations to determine the optimal type [18–21]. With regard to suspensory devices, two commonly observed phenomena with fixed suspensory devices were the “bungee cord effect” and the “windshield wiper effect,” secondary to the longitudinal motion and transverse movement created by the gap between the graft and the fixation, respectively [18, 24]. Many studies have reported that a greater gap would lead to a greater TW, and therefore adjustable suspensory devices were introduced to overcome this deficit [6, 25, 26]. Although, in theory, adjustable suspensory devices could diminish the disadvantage of fixed suspensory devices, Choi et al. reported no significant difference between these two types of devices in terms of tunnel enlargement as well as clinical outcomes [6]. In addition, Bressy et al. reported insufficiency of tibial graft stability when only adjustable suspensory devices were used [27].

In hybrid tibial fixation using CSIS, interference screws present some well-known disadvantages such as migration, loosening, cyst formation and TW. These might be attributable to the less dense structure of the proximal tibia [8, 24]. Thus, the cortical screw post was frequently applied to augment the stability and strength. Indeed, hybrid tibial fixation did result in stronger initial fixation and less knee laxity compared with interference screw alone; yet, this method did not yield significantly better clinical results [9, 28]. As the development of TW is multifactorial, not yet fully clarified, and inevitable in most cases [8], we emphasize the importance of tibial hybrid fixation for its double guarantee and safety for accelerated rehabilitation. The ASIS method was proposed to afford the advantages of both fixation methods and reduce the subsequent complications. As the interference screws had been reported to be associated with graft migration and loosening, we secured the graft by adding adjustable suspensory device to the tibial side, which could reduce the possibility of graft migration; furthermore, the “bungee cord effect” and the “windshield wiper effect” might be decreased owing to less direct graft-to-bone contact and micro-movement owing to the barrier created by the surrounding interference screw.

Furthermore, we observed that three out of the four patients with TW from the CSIS group were female (age range, 40–48 years). Perimenopausal women were reported to possess lower bone mineral density [29, 30]. Perhaps osteopenia or even osteoporosis could be one of the risk factors which in turn lead to TW. Additionally, none of these patients with TW were reported to be regularly exercising in the past. The lifestyle and the natural process of bone loss among middle-age women might weaken the structure in the proximal tibia, presumably leading to greater percentage of TW compared with other patients. Furthermore, a previous study has reported that the transtibial technique could cause more damage to the bony structure than the inside-out method [31]; nonetheless, all patients included in this study had received the same transtibial technique, which potentially eliminated this concern. Nevertheless, we did not document the bone mineral density in our patients after operation as a routine practice. The correlation between bone density and TW would need further study to clarify.

Interestingly, women were more prone to ACL rupture, as stated in previous studies [32–34]. A recent meta-analysis conducted by Mok et al had evaluated what gender could play on the outcomes of ACL reconstruction and demonstrated that functional scores were better post-operatively in men while re-rupture rate was lower in women [35]. Nevertheless, the incidence of TW was not compared in this study. In the meantime, our sample size was not enough to generate a concrete conclusion. Further study would be necessary to clarify the correlation between gender and TW in the future.

Meanwhile, we attributed the percentage of TW to the loosening of the suture. This could have resulted from the cutting off by the sharp margin of the cortical screw or even the tibial tunnel opening, consequently leading to instability between the graft and the interference screw [10, 11]. By contrast, the ASIS method seemed to overcome this issue by replacing the cortical screw post with an adjustable suspensory device. Moreover, considering the routine usage of four stranded autografts with gracilis and semitendinosus, this technique can be used to obtain grafts of sufficient diameter but possess the potential risk of notch impingement for the narrower notch, especially in female patients. Some studies have reported that the notch impingement could account for the TW [15, 36]; hence, more attention should be paid to notchplasty during the procedure. The visualization of TW only in the LAT view might be attributable to the application of a more anterior translation force than the rotatory force for the tibia under the weight-bearing activity after anatomic single-bundle ACL reconstruction.

Aside from the statistical difference in percentage TW, we had found significant difference in post-operative functional scores such as SANE score and Tegner activity level scale between the groups after adjustment for gender. We could also see the similar trend in IKDC score. Our results were similar to those of many other studies [4–7, 9] which stated TW had no clear correlation with clinical outcomes. Nevertheless, improvement in clinical outcomes within each group before and after the operation was discovered. This finding indicated both methods had provided adequate stability and strength for patients. In addition, we had discovered lower degrees of knee flexion prior to operation in ASIS group. The difference in pre-operative status might affect the post-operative results. As for anterior knee pain, despite the lower risk of developing pain in ASIS group, we did not observe significant difference between the groups. Previous studies have reported anterior knee pain owing to the use of cortical screw post in hybrid tibial fixation [10, 11]. Whether the use of ASIS in hybrid tibial fixation could relieve the pain caused by impingement was still unclear. Further studies are warranted to clarify the relationship.

This study has some limitations. Firstly, the patients included in the study were not randomized. The composition of patients might potentially confound the results. As there was no blinding in the study, the preference of examiner and the expectation of patients could influence the assessment. We had had the examiners blinded for physical examination and performed repeatability test for measurement of tunnel widening in order to decrease the influence. In addition, sex distribution showed a significant difference between the groups. Most of the patients in the traditional fixation group as well as most patients who experienced TW were female. We had applied regression models to cope with the confounding effect of the gender.

In addition, we had only documented the TW right after the operation and at least 1 year after the operation. Hence, we could not determine the long-term influence of each operative technique on TW. Nonetheless, as stated in previous research, our course of follow-up yielded adequate results, as the majority of tunnel change occurred within 6 months after the operation [16, 17].

Another limitation was that we did not use the KT-1000 or KT-2000 arthrometer for objective evaluation. Physical examination such as Lachman test could not be precisely graded when there was no measurement instrument applied. Nevertheless, we focused on postoperative TW, and previous studies have also recommended that the KT-1000 arthrometer be used as a diagnostic tool only, as it is unsuitable for use as an outcome tool [37].

With regard to the choice of imaging, radiography was used instead of computed tomography (CT) in this study to measure TW. A previous study reported that CT could provide more accurate and reliable measurements of TW [18, 38]; yet, several studies have reported that radiography could yield acceptable results [6, 39, 40]. On the other hand, we did not conduct MRI on every patient after the operation. Complication such as re-rupture mainly relies on MRI for diagnosis. As a result, some patients with re-rupture might be missed. Our study could not clarify the correlation between TW and re-rupture. Despite these limitations, the present study may still contribute to provide an alternative choice for the tibial hybrid fixation in ACL reconstruction.

## Conclusion

In our study, less tibial tunnel widening in lateral view radiographs was noticed in ASIS group. Even though the patients in the ASIS group had lower post-operative functional scores after adjustment for gender, similar outcomes of physical exams were observed. There was no concrete correlation between TW and clinical outcomes, which was similar to previous studies. In addition, both groups achieved statistically significant improvement after operation. The proposed method may become an alternative option for ACL reconstruction. Nevertheless, the quality of our study is still limited, and thereby researches with larger sample size and long-term follow-up are necessary to generate a more solid conclusion for further application in the future.

## Data Availability

The authors confirm that the data supporting the conclusions of this study is available within the article.

## Abbreviations

ACL: Anterior cruciate ligament
TW: Tunnel widening
ASIS: Adjustable suspensory device and interference screw
CSIS: Cortical screw and interference screw
ROM: Range of motion
IKDC score: International Knee Documentation Committee score
SANE score: Single Assessment Numeric Evaluation score
AP: Anteroposterior
LAT: Lateral
CT: Computed tomography
